# Professional Practice of Ukrainian Doctors in Germany and Poland—Legal and Ethical Considerations

**DOI:** 10.1007/s10728-025-00526-1

**Published:** 2025-06-07

**Authors:** Helena Hointza, Nikola Biller-Andorno, Michael Leitzmann, Julian Werner März

**Affiliations:** 1 University of Regensburg, Regensburg, Germany; 2 University of Zurich, Zurich, Switzerland

**Keywords:** Access to healthcare, Migration of medical professionals, Refugees from Ukraine, Temporary protection

## Abstract

The ongoing war in Ukraine, which began in 2022, has displaced millions of people, creating immense challenges for healthcare systems in refugee-receiving countries. While temporary protection aims to grant refugees access to medical care, significant structural barriers and ethical shortcomings exist in refugee healthcare. To meet this challenge, the authors propose considering the integration of displaced physicians into the medical care systems of host countries. This solution not only meets the immediate healthcare demands but also leverages the expertise of Ukrainian doctors. The implementation in Germany and Poland exemplifies the current heterogeneity of regulations governing the professional practice of Ukrainian physicians, with individual workarounds such as the possibility of treating fellow Ukrainians while waiting for the approval of the license. From an ethical perspective, the dilemma between the urgent need for additional physicians and ensuring patient safety by thoroughly assessing all doctors’ qualifications is a critical concern. Considering all the analyzed aspects, the authors advocate for harmonizing the regulations across the EU and removing barriers that limit healthcare access for refugees. They further stress the importance of developing comprehensive long-term strategies to ensure sustained healthcare access for Ukrainian refugees.

## Introduction

Since February 24, 2022, Ukraine has been facing a severe humanitarian crisis due to Russia’s full-scale invasion. It has led to over 3.6 million people being displaced internally (as of December 2024) and over 6.9 million people being displaced externally (as of April 2025) [36, 66]. Almost 6.4 million refugees from Ukraine were recorded across Europe on August 19, 2024, with many of them seeking refuge in the EU [66]. The main refugee-receiving countries are Germany, which is currently hosting 1.24 million refugees, and Poland, with over 999,000 refugees [66].

As the significant influx of externally displaced people is challenging for the host countries, the EU activated the Temporary Protection Directive on March 4, 2022, to facilitate adequate care for people fleeing Ukraine [22]. Among other rights, temporary protection guarantees its beneficiaries access to medical care [4], which is urgently needed. Analogous schemes have been introduced in the EFTA countries (Iceland, Liechtenstein, Norway, Switzerland) [30].

Refugees from Ukraine have an increased risk of various health threats, for example, contracting infectious diseases due to suboptimal vaccination rates in Ukraine, crowded conditions in transit and refugee accommodation facilities etc. [56]. Further, they are particularly vulnerable to mental health conditions because of traumatic experiences in war and during transit [72]. A survey conducted among refugees in a transit center in Przemyśl, Poland, found that 53.8% of respondents suffer from severe or very severe anxiety, and 57.3% of them show symptoms of severe or very severe depression, which indicates a high level of psychological distress among refugees from Ukraine [54]. Additionally, refugees who suffer from chronic diseases often don’t have access to adequate treatment during the flight [72], which is particularly relevant as the Multi-Sector Needs Assessment in Poland found that 49% of households include a chronically ill member [53]. In addition, a survey conducted by the European Union Agency for Fundamental Rights among Ukrainian refugees in ten EU states found that only 31% of respondents considered their health as good or very good [31], which implies that many refugees could reach out for medical services. Also, there is an increased need for mother and child health services among the refugee population since most refugees arriving in the EU are women and children [37, 53].

In sum, there is a great need for medical assistance among the refugee population. A survey among over 27,000 refugees from Ukraine conducted by the United Nations High Commissioner for Refugees (UNHCR) between February and October 2023 found that 29% of respondents had an urgent need for healthcare [63]. In addition, 33% of respondents needing healthcare experienced difficulties in accessing it.

The rapid influx of numerous people is challenging for the host countries’ healthcare systems [74]. Prusaczyk et al. estimated that in Poland, for every million people added to the population, 2370 physicians would be needed to provide the necessary healthcare services [52].

Since the refugee population includes many highly educated people, many doctors are likely to have fled to refugee-receiving countries [12]. The question arises as to whether this highly skilled workforce could be strategically employed to strengthen healthcare provision.

This paper aims to examine this question comprehensively. First, the legal situation regarding refugee doctors from Ukraine taking up employment in Germany and Poland will be described. In this matter, those countries are of particular interest, as they currently host the highest number of refugees. Further, various ethical considerations will be discussed, including Ukrainian refugees’ access to healthcare, ethical implications regarding the legal regulations, and the so-called brain drain in Ukraine, which describes the loss of highly qualified workers. Finally, the identified potential for improvement will be outlined, focusing on long-term strategies for integrating Ukrainian doctors into the respective healthcare systems and improving the healthcare situation. Even though the focus of this paper will be placed on two states, its results can also be transferred to other European countries, especially other EU members and EFTA countries.

This article focuses on individuals granted temporary protection following their displacement from Ukrainian territory due to the war after February 24, 2022. With approximately 4.3 million people under temporary protection by February 2025, this group constitutes the majority of those displaced. 98.4% of them are Ukrainian nationals [32]. However, it is important to note that surveys on the situation of displaced persons from Ukraine often do not distinguish between individuals with different legal statuses. As such, available data generally reflects the broader population displaced by the war. References to Ukrainian doctors in this paper pertain specifically to those who left Ukraine following the onset of Russia’s invasion on February 24, 2022.

Although not all individuals fleeing Ukraine meet the strict definition of a refugee under the 1951 Refugee Convention [61], they are commonly referred to as refugees in public discourse due to the nature of their forced displacement and the protection they receive under the EU’s Temporary Protection Directive. The use of the term in this article is thus aligned with this broader, context-specific interpretation.

## Possibilities to Take Up Medical Practice: Legal Regulations in Germany and Poland

### Temporary Protection

The EU activated the Temporary Protection Directive on March 4, 2022, and thus created an EU-wide legal basis regarding refugees’ access to certain rights [25]. Temporary protection is a “protection status with reduced formalities”, intended to prevent the overloading of national asylum systems in case of a mass influx of displaced people from non-EU countries [4, 22]. Its beneficiaries are entitled to a set of rights, inter alia, a residence permit for the entire duration of protection, access to accommodation, healthcare, social welfare, and the right to access employment, as far as compatible with national labor market policies. They do not need to apply for asylum, but are allowed to do so simultaneously. If refugees from Ukraine wish not to register for temporary protection, they can also regularly apply for asylum in their host country [25]. The temporary protection status is valid until March 4, 2026 [27].

Registration for temporary protection is possible, but not mandatory, for all people who had their residence in Ukraine before or on February 24, 2022, and belong to the following groups: Ukrainian nationals and their family members; non-Ukrainian nationals and stateless persons under international protection in Ukraine and their family members; non-Ukrainian nationals with a permanent residence permit for whom it is not possible to return to their country of origin safely. For Ukrainian nationals who left Ukraine shortly before February 24, 2022, or were on EU territory temporarily before the war began, for example, for holidays or work, registering for temporary protection may be possible as well. People who do not belong to these groups are granted temporary access to EU territory but must then return to their country of origin [26].

Even though temporary protection beneficiaries are entitled to access the labor market [4], physicians from non-EU states must have their vocational qualifications recognized before starting medical practice, as the profession of a medical doctor is regulated in all EU states [24]. While medical degrees obtained in member states are recognized automatically within the EU, each country individually governs the recognition of medical qualifications obtained in non-EU states [23].

The following will describe the regulations governing access to the labor market for medical professionals from non-EU states in Germany and Poland (Table 1). It is important to note that while these regulations are not explicitly tied to temporary protection status, the majority of refugees hold this status, which then serves as the legal basis for their employment.

**Table 1:** Overview of regulations governing the professional practice of Ukrainian physicians in Germany and Poland

	Germany		Poland		
Regular recognition process for physicians from non-EU states	“Approbation” = permanent and unrestricted permission to practice medicine	“Authorization to practice the profession”	“Right to practice medicine without any restrictions”	“Right to practice the profession of a doctor for a specific range of professional activities, time, and place of employment”	
Restrictions regarding medical tasks	None	Restrictions regarding scope of activities; Work under supervision; Max. for 2 years	None	Restrictions regarding scope of activities, and place of employment; Max. for 5 years; Only for physicians with specialization	
Process	Recognition of vocational qualifications; Equivalence assessment, if necessary	Examination of vocational qualifications	Nostrification of vocational qualifications; Medical verification examination; Postgraduate internship; Medical final examination	Getting approval of the Minister of Health; Requesting the right to practice medicine at the local chamber of medicine	
Language requirements	Level C1 in German	Level C1 in German	Passing language test	Level B1 in Polish	
Other possibilities for Ukrainian refugee physicians to take up work	Working in reception centers for refugees from Ukraine		“Conditional right to practice medicine”		
			Without specialization		With specialization
Restrictions	Work under supervision; Only in reception centers if otherwise the people living there can’t be provided with healthcare;		Work under supervision; Valid for 5 years		Work under supervision for three months; Valid for 5 years
Process	No recognition process; Verification of qualification		Getting approval of the Minister of Health; Requesting the right to practice medicine at the local chamber of medicine		
Language requirements	Need to communicate in the language of the people living in the reception center		Chambers of medicine request “sufficient language skills”; proof of level B1 in Polish until May 2026		
Particularities			Application no longer possible since 25.10.2025; Previously granted practice rights remain valid;		

### Germany

In Germany, physicians from Ukraine who wish to practice their profession need to undergo the usual procedures that apply to citizens of non-EU countries, with one exception.

There are two possibilities for doctors from non-EU countries to get a professional permit in Germany. One option is to acquire the “Approbation”, the permanent and unrestricted permission to work as a physician in Germany. To apply for it, the State Medical Chamber in the federal state the applicant wishes to work in must verify the equivalence of their professional qualification to the medical training in Germany. In some cases, it is sufficient to submit all documents with a translation into German to get the recognition of vocational qualifications [34]. However, if qualifications are not deemed equivalent or essential documents are missing, it may be necessary to pass a knowledge examination in the German language, comparable to the final exam of the German medical studies [10, 15]. Further, the Federal Medical Regulations state that it is necessary to have “the language skills that are necessary to practice medicine” [15]. In 2014, the Conference of Health Ministers decided that physicians needed to prove professional language skills at the level C1 by passing a professional language exam, although each federal state can determine whether the exam is necessary [1]. The process of acquiring the “Approbation” is very lengthy. Usually, between 15 months and three years pass between the application and the granting of permission to practice medicine [59].

If the applicant cannot meet the requirements to apply for the “Approbation”, or to bridge the time until the application is approved, it is possible to work as a physician without recognition but only with “authorization to practice the profession” for a maximum of two years. In this case, the scope of tasks the applicant is allowed to do can be restricted, and they must be supervised by a physician who is holding the “Approbation” [15]. To get the “authorization to work without Approbation”, the applicant needs to submit the same documents as for the “Approbation” and prove German skills at level C1, but there is no equivalence assessment [9].

Further, there is another regulation that is of interest in this context. Following §105d of the Act on the Residence, Economic Activity, and Integration of Foreigners in the Federal Territory, Ukrainian doctors are allowed to treat Ukrainian refugees in reception centers if there aren’t enough doctors to guarantee sufficient health care for those living there. Naturally, physicians must be able to communicate in the language of the people living in the reception center, in this case, Ukrainian. In this setting, they are allowed to work even without further recognition. They only need to verify their qualifications during an expert interview with a doctor appointed by the competent authority. However, these physicians are only permitted to work under the supervision of a licensed doctor [2].

To sum up, getting permission to work as a doctor in Germany is usually very lengthy, and Ukrainian physicians can't start practicing medicine in a timely manner. Following a media report by the German newspaper “Welt am Sonntag”, at least 1,674 doctors from Ukraine have applied for the “Approbation” since February 2022, but only 187 physicians have been authorized to practice medicine since. Accordingly, over 1,400 trained doctors are still waiting for the authorities to process their applications [59]. The only exception could be to work in a reception center under supervision, but there is currently no official data on whether any Ukrainian doctors are working under these conditions without having gone through the recognition process.

### Poland

Doctors from non-EU countries seeking to work in Poland have two options. If they already have a specialization, they can apply for the “right to practice the profession of a doctor for a specific range of professional activities, time, and place of employment”, which allows its holder to work under determined conditions. Acquiring this right to practice involves two steps: obtaining approval from the Minister of Health, followed by applying for a license to practice medicine through the local medical chamber. A full recognition process is not required. However, in this case, physicians are not recognized as specialists. Applying for this work permit requires a certificate confirming that the physician will be employed in a specific medical facility and will only have a scope of practice limited to particular tasks [39]. Further, original documents confirming vocational qualifications, with additional translations by a sworn translator, must be submitted. Since October 25, 2024, applicants must also submit a certificate of Polish language proficiency at level B1. Those who acquired the “right to practice the profession of a doctor for a specific range of professional activities, time, and place of employment” before need to provide a language certificate until May 1, 2026 [44]. This right to practice the profession is valid for five years and cannot be renewed [39].

The other option is to apply for the “right to practice medicine without any restrictions”. This requires a diploma nostrification at one of Poland's medical universities and passing the Medical Verification Examination and the Medical Final Examination conducted by the Center for Medical Examinations in Lodz. Further, applicants must complete a postgraduate internship in Poland for 13 months, unless they have already completed a comparable internship during their education [42]. This pathway also requires the submission of original documents, accompanied by certified translations prepared by a sworn translator. Moreover, it is necessary to prove proficient Polish language skills by passing a language test conducted by the General Medical Council [3]. The test consists of written, oral, and practical components designed to assess the candidate's ability to communicate effectively in medical contexts [46].

Until October 24, 2024, Ukrainian doctors were eligible to apply for the “conditional right to practice medicine”. This option was introduced as an exceptional measure in response to the Ukraine crisis. Although this pathway is no longer available, those who obtained the authorization before the deadline are allowed to continue practicing under its terms [41]. Accordingly, this pathway will also be subject to analysis.

The process of acquiring the “conditional right to practice medicine” was divided into two stages. First, the applicant needed to acquire the Health Minister’s approval, and second, they needed to request the right to practice medicine at the local chamber of medicine. A simple copy of the documents confirming professional qualifications was sufficient for the application if the originals were unavailable. The latter need to be submitted within six months after the end of the armed conflict on Ukrainian territory. These documents also need to be translated by a sworn translator. No language skills needed to be proven, although the medicine chambers requested sufficient Polish knowledge [40, 43, 45]. However, the Polish government decided in October 2024 that those who keep practicing under these conditions must provide proof of Polish language proficiency at level B1 by May 1, 2026 [44].

The “conditional right to practice medicine” allows those who have completed medical studies but do not hold any specialization to work under the supervision of a specialist physician [45]. Ukrainian doctors who additionally have acquired a specialist qualification need to be supervised by a specialist physician for three months and are allowed to work independently afterwards [43]. In both cases, the “conditional right to practice medicine” is valid for five years. If Ukrainian physicians desire to continue practicing afterwards, they must apply for the “unrestricted right to practice medicine”.

Poland has established special modalities to simplify bureaucratic procedures for Ukrainian doctors who wish to work. As a result, by February 2024, almost 4000 doctors from Ukraine, including dentists, had received the Minister of Health’s permission to practice medicine in Poland, which allows them to apply for the right to practice medicine in their local chamber of medicine [57]. However, it is not clear how many of them are actually employed.

By activating the Temporary Protection Directive, the EU has laid the foundation for Ukrainian doctors to take up work. However, implementation of measures is the responsibility of the individual member states. As a result, each country handles the recognition of medical qualifications differently. In Germany, the recognition process is lengthy, resulting in only a few doctors from Ukraine working. Poland, on the other hand, has established significant bureaucratic simplifications for doctors from Ukraine, leading to almost 4000 doctors receiving a work permit from the Minister of Health, the first of two steps in acquiring the right to practice medicine.

## Ethical Considerations

### Ukrainian Refugees’ Access to Healthcare

Aid measures needed to be established quickly and unbureaucratically to provide healthcare to millions of people fleeing Ukraine, especially as the health service delivery situation in host countries around Europe is tense [74]. Evaluating existing care structures, focusing on ethical matters, is necessary to ensure refugees have adequate access to healthcare.

The right to the highest attainable standard of health is enshrined in Article 12 of the International Covenant on Economic, Social and Cultural Rights as one of the fundamental human rights [5]. The United Nations (UN) Committee on Economic, Social and Cultural Rights defined the dimensions of availability, accessibility, acceptability, and quality of healthcare as the essential elements of the right to health [65]. These dimensions should serve as a basis for the analysis of the Ukrainian refugees’ access to healthcare in Germany and Poland, as they reflect the most essential aspects of the subject.

Illingworth et al. argue that “the right to health establishes moral obligations on states not only toward their citizens but also toward people in other nations” [35], implying the necessity of ensuring medical care for refugees. Generally, refugees enjoying temporary protection in the EU have the right to medical care. However, each EU state has implemented the aspects enshrined in the Temporary Protection Directive individually, so the regulations differ within the EU. Consequently, refugees’ experiences may vary throughout the EU. To illustrate the situation, important dimensions of Ukrainian refugees’ access to healthcare in Germany and Poland will be highlighted by way of example, as those countries host around one-third of all refugees from Ukraine in Europe.

It is important to note that the following analysis offers only a limited insight into the situation, as the accessibility of healthcare is influenced by a multitude of factors that cannot be fully captured within the scope of this discussion. Additionally, the availability and quality of data present further limitations. Existing studies frequently rely on small sample sizes or treat refugees as a homogeneous group, without differentiating between their experiences across various host countries.

### Availability

Germany generally provides strong healthcare availability, as evidenced by its high ratio of 4.5 doctors per 1,000 inhabitants, surpassing the EU average of 4.2 [49]. Furthermore, the annual health report published by the Organisation for Economic Co-operation and Development (OECD) indicates that the country faces few unmet healthcare needs, and waiting times for medical treatment are rather short [11, 49]. In Poland, the number of doctors per 1000 inhabitants amounts to 3.5, which is below the EU average. The rate of unmet healthcare needs is 3.6%, with long waiting times being the most frequently cited cause [49]. These statistics suggest that the availability of healthcare services in Poland lags that of Germany. However, both countries face an increasing shortage of medical professionals [38, 78], and the income of many additional patients could aggravate this situation. In the UNHCR survey mentioned above, long waiting times were the obstacle mentioned most frequently (67%) among those who had problems accessing healthcare [63].

As many refugees face language barriers in their host countries [28, 31], the availability of Ukrainian and Russian-speaking doctors is of interest. In a survey conducted by UNHCR, 14% of respondents stated to speak Polish and 2% to speak German [63]. These data indicate that only a few refugees are likely to speak their host country’s official language. Followingly, it might be helpful to consult Ukrainian- and also Russian-speaking physicians, as 82% of respondents to the UNHCR survey stated to speak Russian as well [63]. Davitian et al. found that many participants of their study consulted Russian-speaking physicians in Germany due to language barriers [28]. This aspect might be particularly important in medical specialties like psychiatry or pediatrics, where strong communication skills are crucial. However, finding physicians who speak Ukrainian or Russian can be difficult, as doctor-searching tools often do not offer information about the languages spoken in hospitals and medical offices.

### Accessibility

Data collected by the World Health Organization (WHO) and Statistics Poland among refugees in Poland show that over 90% of the participants who needed healthcare services 30 days before the interview were able to get them [76]. Rolke et al. also found that participants of their study had generally positive experiences in accessing healthcare in Germany [55]. These data imply that healthcare is generally accessible. Nevertheless, it is crucial to assess the barriers refugees face in accessing medical services to further improve healthcare for this vulnerable group.

#### *Information Accessibility*

According to a UNHCR survey, 33% of respondents needing healthcare experienced difficulties in accessing it [63]. Besides long waiting times, as described above, the obstacle mentioned most frequently was the language barrier, indicated by 29% of those who had difficulties accessing healthcare. Additionally, a survey by the European Union Agency for Fundamental Rights found that 47% of respondents encountered language barriers in accessing healthcare [31]. A survey conducted by the WHO and Statistics Poland had similar results [76]. In that survey, 50% of respondents mentioned the information barrier, defined as a lack of information, language, or cultural barrier, as an obstacle they faced in accessing healthcare.

The language and information barrier can affect refugees’ ability to access healthcare on many levels. It can lead to them not getting all the information they need, thus affecting their utilization of medical assistance. For instance, to get the specific healthcare service someone may need, it is necessary to understand the structure of the healthcare system in the country they seek refuge in. Patients need to know which institution is responsible for the kind of service they need, if a referral from another physician is necessary, how to get an appointment, and how long the waiting time will be. Davitian et al. found in their study among Ukrainian refugees in Germany that difficulties in understanding the healthcare system posed a major hurdle in accessing healthcare [28]. Further, refugees need to know about their insurance status and the scope of medical services their insurance covers. The language and information barrier can cause insecurity about financial coverage through insurance, which, in the worst case, might lead to refugees not using medical services despite needing them.

Government institutions around Europe offer official websites that provide information about the respective healthcare systems and access to insurance and healthcare in the Ukrainian language. However, only 45% of the respondents in a UNHCR survey indicated using organization or government websites as an information source [62]. Accordingly, these websites are likely not to reach a significant share of the refugee population. Also, among those using governmental websites, the feedback is heterogeneous. The WHO and Statistics Poland behavioral insights research found that many refugees in Poland consider finding information about medical services on those websites as challenging [76]. On the other hand, in a survey conducted by the German Federal Ministry of the Interior and Community, 92% of respondents who had visited the help website Germany4Ukraine.de were able to find all the information they needed, but only 33% of respondents had accessed it [33]. Most refugees (55%) prefer social media as a source of information instead [62].

The language barrier can be an obstacle regarding organizational aspects of accessing healthcare and during the utilization of medical services. Communication is limited when there is no common language between patient and physician, leading to an incomplete assessment of the patient’s concerns and symptoms and to wrong treatment decisions. Also, not understanding their diagnosis and the physician’s treatment intentions can lead to discontent with healthcare among patients [8]. In addition, lack of communication deprives the patient of the possibility to decide about further treatment procedures based on informed consent. This principle allows the patient to decide about treatment options after having received all relevant information from the attending physician [77]. When patients cannot understand medical information, they depend on the physician’s decision.

Further, in Germany and Poland, it is set by law that all patients are entitled to be informed about their medical conditions, examinations, and treatment in detail and in an understandable manner [6, 16]. However, regulations on handling language barriers in medical settings differ in the respective countries. In Poland, there is no precise legislation about the procedure in this case. The doctor or hospital is not obligated to take care of interpreter availability [51]. If the patient decides to engage a professional interpreter, they need to cover the expenses. In Germany, the law states that the attending doctor must engage an interpreter. However, it does not mandate a professional interpreter, allowing family members or present medical staff to translate as so-called ad hoc interpreters. This term refers to people who are not trained to interpret professionally but have the necessary language skills and are available right away. Usually, patients cannot claim financial coverage for professional interpretation services, although there are exceptions for refugees in some cases [16]. These regulations make it difficult for refugees to use professional interpretation services, as the costs can be high.

#### *Economic Accessibility (*= *Affordability)*

For many refugees, the financial burdens of healthcare are an obstacle to accessing it. In a UNHCR survey, 21% of those who experienced difficulty accessing healthcare stated they could not afford it [63].

In Poland, refugees from Ukraine are entitled to health insurance in the same way as Polish people are [47]. The government states that both Ukrainians and Polish citizens enjoy the same access to healthcare [48]. In Germany, regulations differ for refugees “in need”, which means they don’t have a regular income or financial reserves, and those who are not in need. The former have access to statutory health insurance, and the latter can join the voluntary insurance scheme in the statutory health insurance system (GKV), which means they need to contribute to the insurance [14].

All these regulations are based on the Temporary Protection Directive [31], but financial access to healthcare is not regulated uniformly in the EU. Also, public health insurance does not cover all healthcare services that might be necessary, such as specific medication or medical remedies, which can lead to financial hurdles in accessing healthcare.

#### *Non-Discrimination*

Following the principle of non-discrimination inherent in the right to health, healthcare services provided to refugees must adhere to the highest attainable standards of quality, ensuring equitable and effective care irrespective of legal or migration status [65]. The WHO states that “discrimination in health care settings takes many forms and is often manifested when an individual or group is denied access to health care services that are otherwise available to others” [73]. Given the previously discussed barriers to healthcare access for refugees from Ukraine, it is likely that they face an elevated risk of experiencing discrimination within healthcare settings. A study about Ukrainian refugees’ access to sexual and reproductive health in five EU states, including Poland, found that they face severe discrimination in this field [18]. These data indicate an increased risk of discrimination against Ukrainian refugees in healthcare. However, further research in this field is necessary to fully understand the scope of this issue, as there is little data on this subject.

### Acceptability

It is necessary to consider cultural peculiarities in the doctor-patient interaction. Physicians need appropriate qualifications regarding cultural differences and especially war-related psychological impairment, as in conflict settings, the prevalence of the latter is particularly high. The WHO estimates that 22.1% of conflict-affected populations suffer from mental disorders [19], and Buchcik et al. have found that 40.5% of surveyed Ukrainian refugees in Germany suffer from severe psychological distress [13]. Additionally, 1.49 million people have been displaced internally within Ukraine due to the armed conflict between Ukraine and Russia along the border of Crimea, which was annexed by Russia in 2014 [29]. Therefore, many refugees have already been suffering from pre-existing trauma [17]. Of course, treatment of mental health disorders, including those attributed to trauma, needs to be undertaken by psychiatrists and professional therapists. Nevertheless, all physicians, especially family doctors, who are often the first point of contact for patients, need to be able to detect mental health disorders to refer patients to adequate specialists.

### Quality

To achieve beneficial health outcomes, healthcare must not only be accessible but also of high quality [75]. In the CEOWorld Magazine’s Healthcare Index, “a statistical analysis of the overall quality of healthcare systems”, Germany ranks 8th and Poland 59th out of 110 countries [68]. Consequently, Germany offers a high-quality healthcare system by international standards, whereas Poland’s system performs at a rather moderate level. However, the concept of “quality of care” is complex and multifaceted, and there is no universal definition for the term [50], which leads to limitations in assessing healthcare quality in Germany and Poland comprehensively within the scope of this paper. Information about specific indicators of the quality of healthcare can be found in the annual OECD report [49] and systematic reviews about the healthcare systems in Germany [11] and Poland [58]. The subjective perception of the healthcare quality among Ukrainian refugees, however, was found to be mostly positive in both countries [55, 76].

The preceding analysis has shown that the EU and the individual member states have already taken many measures to provide medical care for refugees. However, refugees still face several challenges in accessing healthcare, such as long waiting times, financial hurdles, and language and information barriers. In sum, there is room for improvement. This raises the question of the extent to which refugee doctors can contribute to improving healthcare provision for fellow Ukrainians.

### Displaced Physicians Working in Germany and Poland: Ethical Implications

### Poland

The following section analyzes the potential benefits of integrating Ukrainian doctors into the healthcare systems of their host countries, as well as the challenges this approach may present. To systematically evaluate this subject, two possible integration models will be considered. Furthermore, the analysis considers the needs of both the general patient population in host countries and, more specifically, those of refugees from Ukraine.

One potential approach is to grant Ukrainian doctors timely access to the labor market through simplified administrative procedures. In Poland, for instance, they could acquire the “conditional right to practice medicine”. This option is no longer available, as described above. However, the physicians who acquired the “conditional right to practice medicine” are allowed to continue practicing. These doctors are authorized to treat all patients, not only refugees from Ukraine. By addressing the physician shortage outlined above, this measure could enhance the overall availability of medical services. Moreover, their proficiency in the Ukrainian language may improve healthcare accessibility for Ukrainian refugees, particularly by mitigating language barriers that often hinder access to healthcare services. These language skills not only improve communication but also enhance the accessibility of health-related information, thereby supporting patient autonomy. In addition, sharing a common cultural background with Ukrainian patients may make refugee doctors more attuned to cultural nuances, therefore potentially improving acceptability. However, knowledge of the host country’s language is essential to ensure that healthcare services provided by refugee physicians are also accessible to the local population. Even though they now must prove language proficiency at level B1 by May 2026, physicians from Ukraine did not need to prove any language skills to apply for the “conditional right to practice medicine” in the first place. Further, it is uncertain if this language level is sufficient to meet patients’ communication needs. This could lead to problems in communication and impair fulfillment of the patient’s right to knowledge about their health status and treatment.

Skilled medical personnel are an essential component of high-quality healthcare [65]. The qualifications of all applicants must be carefully reviewed to ensure they meet the local standards of patient care. Despite the urgency of acquiring additional health personnel, patient safety must be the top priority. It is questionable whether procedures designed to be completed as quickly as possible meet this requirement. Further, it is essential for physicians not only to have medical knowledge and professional competence but also to understand how the healthcare system operates and to be familiar with its administrative procedures. Besides direct patient care, they are responsible for prescribing medication, interpreting clinical findings, and initiating further diagnostic or therapeutic measures. Communication with other health personnel is often necessary to ensure proper care. Further, a basic understanding of insurance policies is also necessary to ensure appropriate and accessible care. This is particularly important when it comes to treating refugees, as their insurance status may differ from usual insurance. A lack of these skills may impair various aspects of healthcare quality, such as patient safety or patient-centeredness, which are seen as core dimensions of quality of healthcare [50]. Followingly, hiring migrant health professionals without additional training in this field could affect the quality of healthcare due to language barriers and a lack of knowledge of the host country's healthcare system, which affects all patients equally.

### Germany

Another approach to improving healthcare provision, considering the significant influx of refugees is to authorize Ukrainian doctors to treat Ukrainian refugees exclusively, without requiring full recognition of their qualifications. This measure would, however, be limited to this specific patient group. In Germany, this approach was partially implemented in the form of §105d of the Residence Act, as described before. This measure's advantage lies in the prompt availability of Ukrainian-speaking physicians without the need for prolonged recognition procedures. It could improve patient-doctor communication, thus enhancing healthcare accessibility. The WHO and Statistics Poland behavioral insights research found that respondents frequently consulted Ukrainian doctors in Poland when possible [76], indicating that many patients would make use of such an offer. However, it cannot be guaranteed that the qualifications and skills of a physician will be thoroughly examined. Furthermore, the qualifications of Ukrainian physicians may differ from those of the local staff, which could lead to differences in the quality of care, as described above. Considering the principle of non-discrimination, it is not permissible to let a physician treat patients who fled Ukraine under different, less strict conditions than the host country’s population. All patients must be treated equally, regardless of their origin and nationality. However, in Germany, refugee doctors are only allowed to practice in this setting under the supervision of a licensed physician. A prerequisite for this arrangement is that the doctors share a common language, such as English. If this requirement is implemented appropriately and close supervision is ensured, Ukrainian physicians could contribute to the care of refugees living in reception centers without recognition, if the need arises.

To meet the identified challenges and fully tap into the potential of refugee doctors, initiatives should be promoted that support Ukrainian physicians in learning the host country’s language and understanding the structure and functioning of its healthcare system. For example, the WHO, the Polish Ministry of Health, and the Centre of Postgraduate Education in Warsaw have developed such a course to support doctors from Ukraine in integrating into the Polish healthcare system [74]. Furthermore, a systematic evaluation should be undertaken to examine how the integration of Ukrainian doctors is progressing in practice and to what extent the expected challenges are materializing.

### Brain Drain and WHO Code of Practice

Ukraine had to deal with the phenomenon of “brain drain”, which describes highly educated people leaving the country to live and work abroad, already before Russia’s invasion on February 24, 2022 [60]. However, the situation got aggravated drastically with the beginning of the war, as 6.9 million people left the country by April 2025, which is around 16% of the population of 43.8 million people Ukraine had in 2021 [66, 69]. In a representative survey conducted by the German Federal Office for Migration and Refugees among over 11,000 people who fled from Ukraine to Germany, 72% hold a degree of tertiary education, which is notably above the average level of 50% in the Ukrainian population in general [12]. These data imply that many highly educated people left Ukraine due to the war, which means a significant loss of specialized workforce for the country.

In the following, some thoughts on the migration of highly educated people, including doctors and other medical professionals, will be discussed, considering the “WHO Global Code of Practice on the International Recruitment of Health Personnel”, which is a voluntary code of practice for the WHO member states [70].

Even though the emigration of the healthcare workforce means a loss for their home country, it is every person’s, thus every medical professional’s, free choice to leave their country of origin to migrate to another state [70]. Accordingly, the consequences of the “brain drain” in Ukraine do not affect physicians’ freedom of choice to leave the country. Furthermore, the shortage of healthcare workers in EU states has been a challenge already before the mass influx of refugees [71]. Even though physicians among the refugees can contribute to relieving the tense situation in the healthcare sector, they will not compensate for the shortage of medical staff in general. Instead, all countries must create structures to train enough healthcare personnel themselves to ensure high-quality healthcare for all patients [70]. Finally, refugee-receiving countries can contribute to mitigating the effects of the currently accelerated brain drain in Ukraine by giving Ukrainian physicians the possibility to work, so they can consolidate their skills and expand their medical knowledge, which can be helpful if they decide to return to Ukraine after the war is over [74].

## How to Move Forward

It is uncertain how long the armed conflict on Ukrainian territory will continue, forcing people to leave their homes to seek refuge in other countries. Therefore, the existing aid measures should be assessed steadily to improve the unbureaucratic access to help for those who need it due to this terrible war.

The preceding analysis has demonstrated that doctors from Ukraine can provide valuable support to the healthcare systems of their host countries by contributing their knowledge and expertise. Their integration reinforces the health workforce and helps alleviate physician shortages. In addition, their proficiency in the Ukrainian language particularly enhances access to healthcare services for refugees from Ukraine. However, to ensure high-quality healthcare and treatment free of discrimination, the identified ethical concerns must be addressed. Consequently, policies, whether at the national or EU level, must ensure that refugee doctors possess qualifications that meet host country standards, demonstrate sufficient language proficiency, and have an adequate understanding of the respective healthcare system. To assess the effectiveness of the current regulations, it is essential to examine how well Ukrainian doctors integrate into the host country's healthcare system, whether they are able to navigate clinical routines, and to what extent theoretical concerns manifest in practice. A systematic analysis enables the improvement of the regulations and provides valuable lessons for the future.

In addition, the authors suggest alignment of regulations within the EU. By activating the Temporary Protection Directive, the EU generally enables refugees from Ukraine to work in their host country without a lengthy asylum procedure. However, as the medical profession is regulated throughout the EU, recognition of professional qualifications is necessary. It is desirable to harmonize the relevant policies of individual member states to enhance transparency and fairness in the recognition process across the EU, especially considering the automatic recognition of medical degrees within the EU [23]. As, following Article 168 of the Treaty on the Functioning of the European Union (TFEU), governing public health policies is the responsibility of the individual member states, the EU's scope for action in this field is limited [7]. Nevertheless, the EU could promote the harmonization of policies as “the Union shall encourage cooperation between the Member States […] and, if necessary, lend support to their action” [7]. In addition, the European Council can give recommendations on public health matters based on Article 168 of the TFEU [7]. The EU should leverage these instruments to promote greater harmonization of policies governing the professional practice of Ukrainian doctors.

Looking at health services, many measures have already been taken to provide comprehensive healthcare to refugees from Ukraine. However, the preceding analysis has revealed that significant challenges remain in this area. While doctors from Ukraine can make a valuable contribution to addressing these challenges, their integration into the host countries’ healthcare systems alone will not be sufficient to resolve them fully. Therefore, the aid measures should be systematically evaluated and continuously improved. Particular attention should be paid to addressing financial hurdles in accessing healthcare and reducing information barriers. Further research on the refugees’ health literacy and the subjective usefulness of existing information sources needs to be conducted to improve the distribution of information about healthcare. Current data suggest that information distribution via social media should be expanded, as that information source is popular among refugees. Moreover, instances of discrimination should be systematically assessed to prevent discrimination and ensure equitable access to healthcare services.

Finally, to sustainably improve healthcare in the future, the ongoing shortage of medical professionals must also be addressed. Even though physicians among the refugee population can support their host countries’ healthcare systems, they will not solve this problem. Therefore, all countries must train enough health personnel to guarantee high-quality healthcare delivery for all their patients.

### Long-Term Strategy

In a UNHCR survey conducted among refugees in various host countries in Europe between December 2022 and January 2023, 12% of respondents indicated they had plans “to return [to Ukraine] permanently in the next three months”, and 65% responded they “hope to eventually return to Ukraine in the future”, whereas 18% had not decided yet, whether to return [64]. 5% reported not having plans to return to Ukraine. Following these data, most refugees are not planning to stay in their host country for the long term.

The temporary protection status is valid until March 4, 2026. Usually, temporary protection can be extended for up to three years, which was over in March 2025, but due to the ongoing war in Ukraine, the European Council extraordinarily decided to extend it further [27]. It is now essential to establish new legal frameworks to govern the continued stay of refugees in the EU after the expiration of temporary protection. The EU has not presented a coordinated strategy on this matter yet. However, some member states have already introduced measures to address this demand [67]. Poland, for instance, has introduced another residence status option for Ukrainian refugees. Since March 2025, those with at least one year of continuous temporary protection status are permitted to apply for a temporary residence card, which will be valid for three years [21, 67]. Wagner and Grama argue that a EU-coordinated strategy to exit temporary protection gradually would “help prevent fragmentation and ensure equal treatment across countries, reducing the risk of secondary movements and disparities in protection standards” [67]. A corresponding framework must consider the needs of both those who wish to return to Ukraine and those who seek to establish a long-term future in their host country [67]. Regardless of how temporary protection is further handled, refugees also have the possibility to apply for international protection, if they have not already done so, or to remain in the EU according to national immigration policies [20].

It remains to be seen how many doctors will be among those who stay in their host country in the long term. In addition to their residence status, the working conditions of refugee doctors may also change, depending on the host country. The “conditional right to practice medicine”, which was granted to Ukrainian refugee doctors until October 2024 as part of Poland’s emergency response to the Ukraine crisis, is limited to a maximum duration of five years and cannot be extended. The same applies to those who have acquired the “right to practice the profession of a doctor for a specific range of professional activities, time, and place of employment”. Followingly, physicians who seek to work in Poland permanently must apply for unrestricted work permission if they have not by then. In Germany, Ukrainian doctors had to apply for the Approbation, which represents the unrestricted work permission in Germany, in the first place. For those who have acquired the Approbation, there will not be any changes regarding their right to practice medicine. Since working in refugee accommodation without recognition is only permitted when medical care cannot be provided otherwise, this option will no longer be available once that condition is no longer met.

## Conclusion

The EU and its member states have implemented a range of measures to ensure access to healthcare for refugees from Ukraine. However, the provision of care to such a large and vulnerable patient population presents significant challenges, and certain healthcare needs remain unmet. The integration of displaced physicians into host country healthcare systems represents a promising strategy to help mitigate these challenges. Nevertheless, to guarantee high-quality care for both the host population and refugee patients, legal and ethical challenges must be thoroughly identified and systematically addressed.

## Data Availability

No datasets were generated or analysed during the current study.
